# Supplementary material for: “Know your epidemic, know your response”: Epidemiological assessment of the substance use disorder crisis in the United States
Source: PLoS One. 2021 May 26;16(5):e0251502. doi: 10.1371/journal.pone.0251502 (PMC8153501; doi:10.1371/journal.pone.0251502)
Supplement: S1 Text — (DOCX) [file pone.0251502.s001.docx]

**S1 Text.**

1. **Methods Supplement**
   1. **Cause of death classification and substance included in our analysis**

Data included in our study was retrieved from the CDC from restricted-use vital statistics micro-data files, for the period of January 2005 to December 2017, and included the date and county of death, demographic and socioeconomic characteristics of individuals (sex, race, age, marital status, and educational level) and the International Classification of Diseases, 10^th^ Revision (ICD-10) code for the cause of death [1]. We extracted information about drug overdose deaths for individuals aged 5 to 84 years from ICD-10 codes for unintentional substance poisoning (cause of death codes: X40, X41, X42, X43, X44) to estimate substance use disorder (SUD) mortality rates. These ICD-10 codes included deaths caused by the following substances: heroin, methadone, cocaine, other opioids, synthetic narcotics, and unspecified narcotics.

- 1. **Description of Mentally and Physically Unhealthy Days Estimation**

The average number of days per month of self-reported physical and mental distress, derived from the County Health Rankings & Roadmaps program from 2010 to 2017, were collected from questionnaires conducted by phone interview. The respondent answered the following two questions: “*Now thinking about your physical health, which includes physical illness and injury, for how many days during the past 30 days was your physical health not good?”* and “*Now thinking about your mental health, which includes stress, depression, and problems with emotions, for how many days during the past 30 days was your mental health not good?”*, as proxies for physical and mental health conditions of the respondent [2].

- 1. **Stratified random sampling**

We conducted stratified random sampling, using strata given by the year and county of death occurrence, to reach feasible computational resources for our hierarchical regression analysis. Stratified random sampling has been applied in previous research involving hierarchical models for estimating community-level associations [3, 4].The goal of this sampling technique when applied to large scale spatiotemporal applications is to retain the characteristics of the complete (full population) data set in the study sample (i.e., retain generalizability) while reducing the standard error compared to a simple random sample [4]. The characteristics of the stratified sample are described in the S1Table.

- 1. **Generalized Additive Models Regression**

The community-level covariates were averaged by county from annual data for 2010 – 2017. We included self-reported frequency of days under physical and mental distress per month, and we calculated the percentages of excessive adult drinking, tobacco consumption, children living in poverty, and uninsured at the county level; each study subject was assigned the corresponding county-level value of these variables.

We also conducted three separate logistic regression GAM mixed models, In addition to our main logistic regression analysis. We investigated for interactions between the average number of mental and physical unhealthy days and each of age, sex, and race groups.

- 1. **Clustering Analysis and the Relative Risk Estimation Analysis**

Our clustering analysis was conducted using SaTScan[5], in which the identification of hotspots was achieved by using likelihood ratio and t-tests for testing potential clusters against the null hypothesis of a constant risk in which the expected number of cases is proportional to the total number of deaths. In addition, for the Relative Risk (RR) estimation, the INLA analysis included a BYM model that incorporates spatially correlated random effects using a conditionally auto-regressive structure. A second unstructured random effect was included in the model, and its variance component was modeled with a diffuse gamma prior distribution. Finally, we added the averaged community- level covariates described in the previous section of each county as covariates in the model to quantify the impact of socioeconomic and health risk factors on the RR at the county level.

1. **Results Supplement**
   1. **Substance Discrimination Analysis Results**

The result from our substance discrimination analysis (Supplementary Figure 1) shows several substances that prominently contributed to SUD mortality rates over time, starting with heroin and opioids (mainly prescription opioids) early in the epidemic, and synthetic opioids in the later phase of the epidemic. Synthetic opioids, which were the main driver of SUD-related deaths during 2017, were 45·19% of the total SUD-related deaths, followed by heroin (23·96%) and other opioids (19·43%). There was variation in temporal trends between the two demographic groups with the highest mortality rates. The SUD-related mortality rate for White males was consistent with the general population. Conversely, Black males had higher percentages of SUD-related deaths associated with synthetic opioids (48·29%), heroin (26·8%), cocaine (7·61%), and lower rates due to other opioids (11·35%).

- 1. **Mentally and Physically Unhealthy Days Interaction Model Results**

Mentally unhealthy days had a significant effect on the odds of SUD-related death in individuals aged 20 – 54 years, while physically unhealthy days was associated with increased odds in individuals aged 35 years and older. There was no evidence that the statistical associations between mental and physical health and SUD-related deaths varied by sex, and race (S2 Table), with exception of the mental health - race interaction. This means that mental and physical distress was equally significant for females and males, and we only found a non-significant association of mental distress on the odds of SUD-related deaths among the black population.

- 1. **Distribution of the temporal trending of Relative Risk in the SUD identified hotspots**

The temporal RR estimation analysis from our spatiotemporal INLA analysis show that hotspot areas experienced an average RR percent change of +12.39% compared to non-hotspot areas, which had a decline in RR of 11.28%. Additionally, the distribution of counties where RR was increasing more than 80% resulted in 161 counties (5.4% of all counties), located in the Northeast (68 counties), South (49 counties), Midwest (39 counties), and West (5 counties) respectively.

Finally, the Figure 4 (B) (in our study) describes the temporal trends of SUD-related mortality risk by estimating the percent change between the RR for the first semester of 2005 and the RR for the last semester of 2017. There was a substantial increase in RR for the Northeast region, with an average percent change of +64·78% compared to the national average of -7·20%, which suggests a spatial concentration of the epidemic in the Northeastern region with an increasing trending by the end of 2017.

- 1. **Effects of the selected covariates on the SUD related Death Relative Risk Distribution**

We found that mental and physical distress contributed significantly to increased risk of death caused by SUD (RR=1.40 and 1.28, respectively). This also is shown in the difference between the mentally and physically unhealthy days within hotspots, with a weighted average of 4.02 and 4.20 days, respectively, compared to the non-hotspot areas, with 3.57 and 3.75 days, respectively. Moreover, excessive alcohol consumption did not have a significant link to higher RR of SUD-related mortality (RR: 1.004, 95% CI: 0.998 – 1.011), whereas tobacco consumption (RR: 1.011, 95% CI: 1.004 – 1.018) was significantly associated with higher RR of SUD-related mortality at the county level.

1. **Implications of the Socioeconomic Status impact on SUD related mortality rates**

The assessment of the effect of socio-economic status (SES) on the individual odds of SUD-related deaths indicated an inverse association with the proportion of uninsured population and children living in poverty. The relationship of addiction, low literacy, and SES is still not clear [6]. Although low SES is an accepted risk factor for addiction, there are findings of an increasing risk of substance consumption in high income and educated families, especially in young adult population [6, 7]. Reasons behind this are attributed to higher pressure in adolescents of high-income families for educational accomplishments, and greater acceptance of substance consumption by highly educated parents that could trigger anxiety, depression and psychological illness that prompt addiction disorders, and access to prescription opioids [6, 8]. Moreover, according to the CDC, heroin use has increased from 2011 to 2013 in all demographic groups, but especially in families with incomes higher than 20,000 USD year [9]. Therefore, main drivers of the addiction disorders especially in adolescents and young adults are likely related to mental and physical illness that make this population vulnerable to substance abuse more than specific SES characteristics.

For the 34,499 White males aged 30-34 who died by SUD-related causes from 2005 to 2017 in the U.S., 22,897 (66·37%) were never married, 6,641 (19·24%) currently married, and 4,564 (13·22%) previously married, with an average monthly exposure to mentally and physically unhealthy days of 3·7. Youngest black adults have higher overdoses by heroin and synthetic opioids for recreational purposes, and older black adults show higher overdoses due to cocaine and prescription opioids exhibiting the aging of the cohort of the previous wave of the addiction epidemic [10]. Similarly, women and older adults showed higher rates of drug overdose due to prescription opioids than younger adults [11]. Differences in the demographics of the addiction epidemic reveals that multiple drug types that caused the outbreak are eventually converging into a significant public health issue due to the mixture of previous waves and the inclusion of new substance in the epidemic like synthetic opioids, which are far more lethal substances [12]. Additionally, 2,752 black males aged 30-34 died by SUD from 2005 to 2017, with 2,090 (75·94%) never married, 457 (16·60%) currently married, and 155 (5·63%) previously married, and an average exposure to mentally and physically unhealthy days of 3·7.

1. **Prevalence estimation of dual-diagnosis and SUD in medicated patients in the U.S.**

Mental health and SUD comorbidity are known as co-occurring disorder or dual diagnosis is a long-known associated illness [13-15]. It refers to a clinical picture that independently meets the criteria for at least one mental health and one substance use disorder [13]. This picture is commonly diagnosed among adolescents and young adult patients with substance use disorders, and it is estimated that 50% of patients with SUD could have symptoms of depression, anxiety, post-traumatic stress disorder, psychosis, or mania, and only 12% of those are likely to receive treatment for both illnesses [14, 15].

Furthermore, we found that an additional average day of physical distress might increase the RR of SUD related mortality by 28%, and this factor was affecting more older adults with a shaper effect in the White population. Around 15% of patients using opioid pain medications become addicted, and 70% of those abusing pain medications are illegally obtained [16]. Physical distress holds a strong link with pain medication dependence, which are related but not equal to substance addiction [16].

1. **Additional considerations of significant high concentration of SUD related deaths in the Midwest areas**

The unusual high burden of SUD-related deaths in this area could be associated to its historical role as corridor of illegal substances into its way to the U.S. market [17]. This region is also characterized by a high flow of illegal synthetic opioids reported by the opioid’s seizures for Ohio, Kentucky, Indiana, and West Virginia, and high rates of average physically and mentally unhealthy days [18].

1. **References**

1. World Health Organization. *International classification of diseases for mortality and morbidity statistics* (10th Revision). 2019.

2. Centers for Disease Control and Prevention (CDC). Behavioral Risk Factor Surveillance System Survey Questionnaire. In: Atlanta Georgia: U.S. Department of Health and Human Services, Centers for Disease Control and Prevention, editors. 2010-2017.

3. Murase H, Nagashima H, Yonezaki S, Matsukura R, Kitakado T. Application of a generalized additive model (GAM) to reveal relationships between environmental factors and distributions of pelagic fish and krill: a case study in Sendai Bay, Japan. ICES Journal of Marine Science. 2009;66(6):1417-24. doi: 10.1093/icesjms/fsp105.

4. Boschetti L, Stehman SV, Roy DP. A stratified random sampling design in space and time for regional to global scale burned area product validation. Remote sensing of environment. 2016;186:465-78. Epub 2016/09/20. doi: 10.1016/j.rse.2016.09.016. PubMed PMID: 30416212.

5. Kulldorff M, National Cancer Institute. SaTScan v9.0: software for the spatial and space-time statistics. 2010.

6. Patrick ME, Wightman P, Schoeni RF, Schulenberg JE. Socioeconomic status and substance use among young adults: a comparison across constructs and drugs. Journal of studies on alcohol and drugs. 2012;73(5):772-82. doi: 10.15288/jsad.2012.73.772. PubMed PMID: 22846241.

7. Altekruse SF, Cosgrove CM, Altekruse WC, Jenkins RA, Blanco C. Socioeconomic risk factors for fatal opioid overdoses in the United States: Findings from the Mortality Disparities in American Communities Study (MDAC). PloS one. 2020;15(1):e0227966-e. doi: 10.1371/journal.pone.0227966. PubMed PMID: 31951640.

8. Whitesell M, Bachand A, Peel J, Brown M. Familial, Social, and Individual Factors Contributing to Risk for Adolescent Substance Use. Journal of Addiction. 2013;2013:579310. doi: 10.1155/2013/579310.

9. U.S. Department of Health and Human Services, Substance Abuse and Mental Health Services Administration, Center for Behavioral Health Statistics and Quality. National Survey on Drug Use and Health 2016 (NSDUH-2016-DS0001). 2018.

10. Dunlap E, Golub A, Johnson BD. The Severely-Distressed African American Family in the Crack Era: Empowerment is not Enough. Journal of sociology and social welfare. 2006;33(1):115-39. PubMed PMID: 18852841.

11. Jalal H, Buchanich JM, Roberts MS, Balmert LC, Zhang K, Burke DS. Changing dynamics of the drug overdose epidemic in the United States from 1979 through 2016. Science. 2018;361(6408):eaau1184. doi: 10.1126/science.aau1184.

12. Fischer B, Jones W, Tyndall M, Kurdyak P. Correlations between opioid mortality increases related to illicit/synthetic opioids and reductions of medical opioid dispensing - exploratory analyses from Canada. BMC Public Health. 2020;20(1):143. doi: 10.1186/s12889-020-8205-z.

13. Hawkins EH. A Tale of Two Systems: Co-Occurring Mental Health and Substance Abuse Disorders Treatment for Adolescents. Annual Review of Psychology. 2008;60(1):197-227. doi: 10.1146/annurev.psych.60.110707.163456.

14. Harris KM, Edlund MJ. Use of Mental Health Care and Substance Abuse Treatment Among Adults With Co-occurring Disorders. Psychiatric Services. 2005;56(8):954-9. doi: 10.1176/appi.ps.56.8.954.

15. Watkins KE, Hunter SB, Wenzel SL, Tu W, Paddock SM, Griffin A, et al. Prevalence and Characteristics of Clients with Co‐Occurring Disorders in Outpatient Substance Abuse Treatment. The American Journal of Drug and Alcohol Abuse. 2004;30(4):749-64. doi: 10.1081/ADA-200037538.

16. Edlund MJ, Steffick D, Hudson T, Harris KM, Sullivan M. Risk factors for clinically recognized opioid abuse and dependence among veterans using opioids for chronic non-cancer pain. PAIN. 2007;129(3).

17. Ciccarone D. The triple wave epidemic: Supply and demand drivers of the US opioid overdose crisis2019.

18. Center for Disease Control and Prevention. Increases in Fentanyl Drug Confiscations and Fentanyl-related Overdose Fatalities. Center for Disease Control and Prevention, 2015 10/26/2015. Report No.
