# Supplementary figures and images for: “Know your epidemic, know your response”: Epidemiological assessment of the substance use disorder crisis in the United States
Source: PLoS One. 2021 May 26;16(5):e0251502. doi: 10.1371/journal.pone.0251502 (PMC8153501; doi:10.1371/journal.pone.0251502)

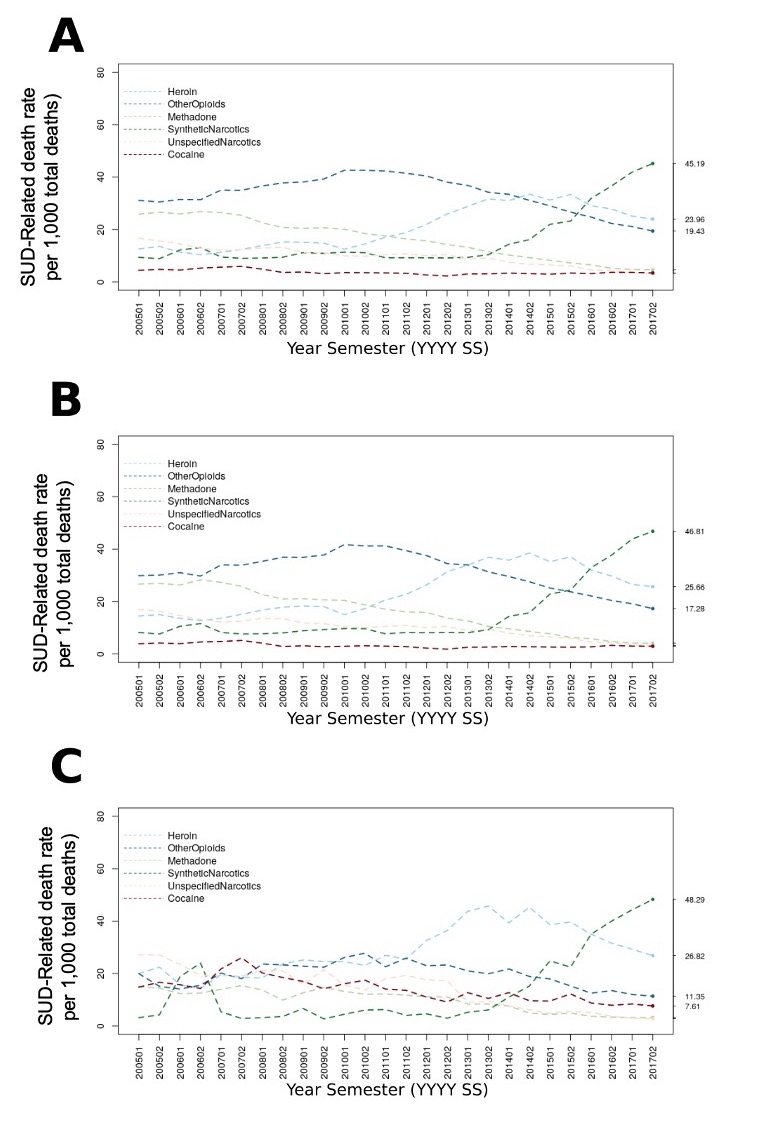

Supplement: S1 Fig — (A) Total population, (B) White males (C), and Black males. (JPG) [file pone.0251502.s002.jpg]

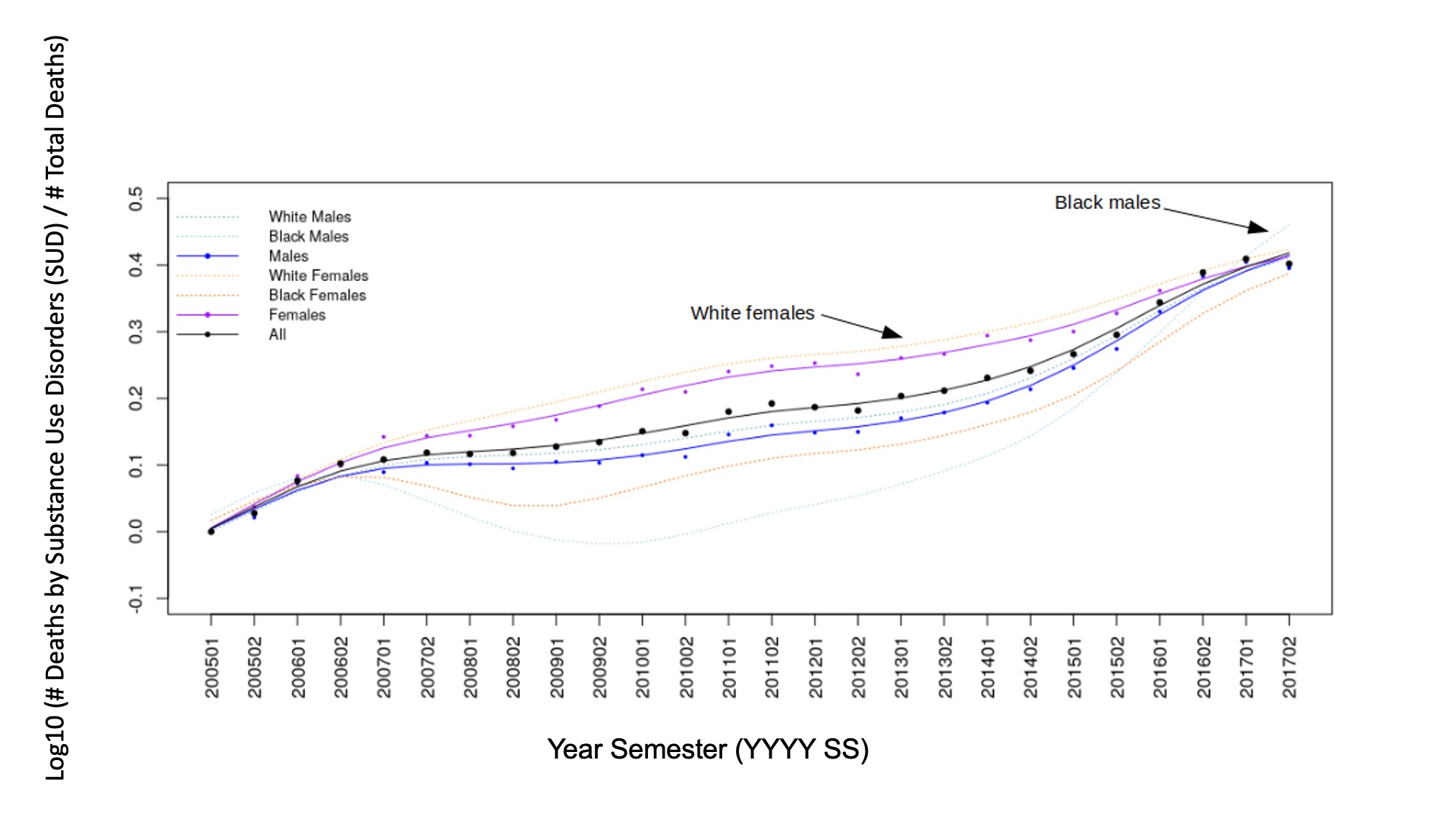

Supplement: S2 Fig — (Log10 (# Deaths by Substance Use Disorders / # Total Deaths)) of the substance use disorder (SUD) epidemic in the U.S. (2005–2017) by semester. (JPEG) [file pone.0251502.s003.jpeg]

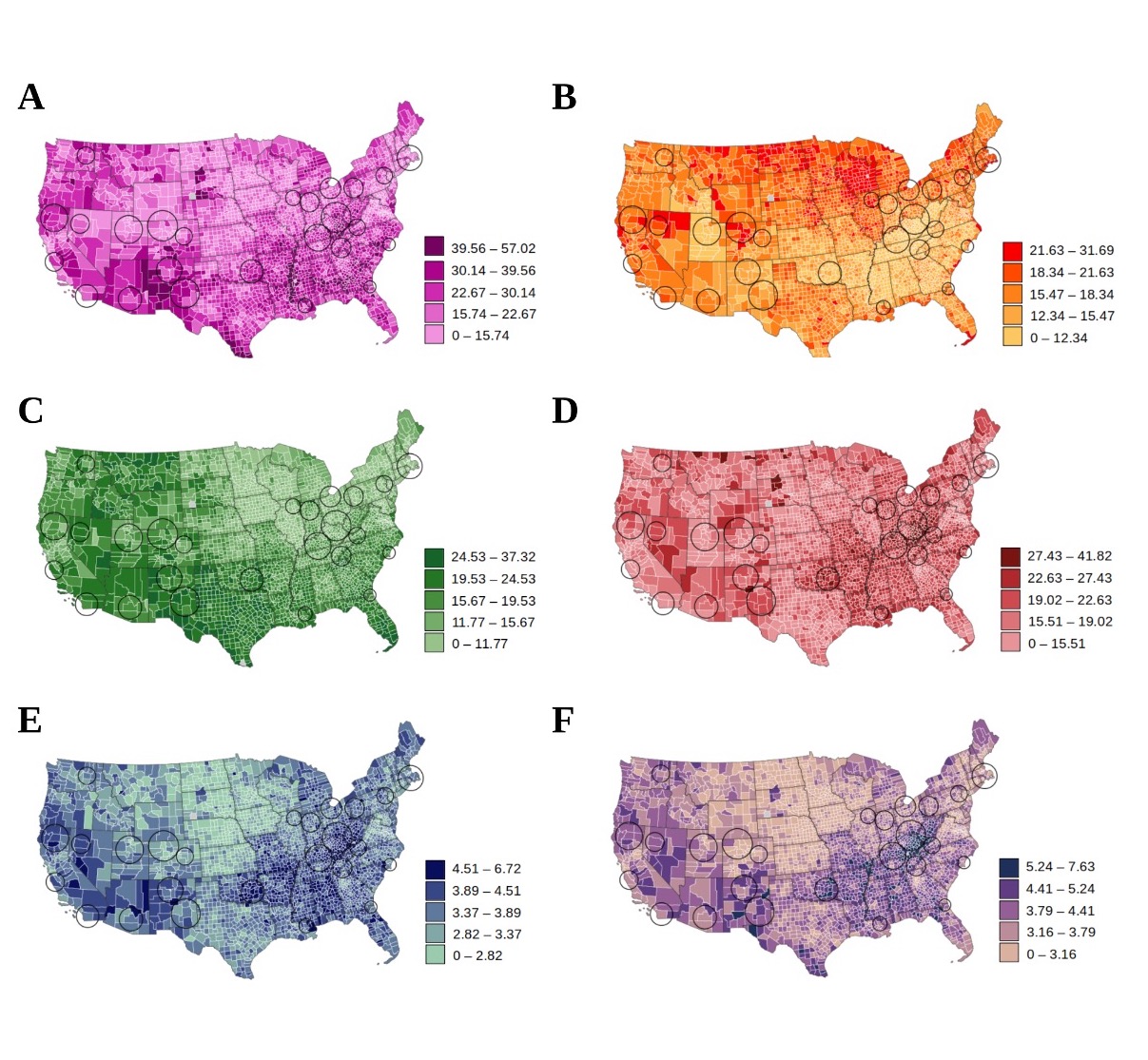

Supplement: S3 Fig — (A) Percentage of children living under poverty, (B) percentage of the adult population that consume alcohol excessively, (C) percentage of the uninsured population, (D) percentage of the adult population that consumes tobacco, (E) average of mentally unhealthy days, (F) average of physically unhealthy days. All values are averaged from 2010–2017 by county. (JPEG) [file pone.0251502.s004.jpeg]
