# Supplementary material for: “Know your epidemic, know your response”: Epidemiological assessment of the substance use disorder crisis in the United States
Source: PLoS One. 2021 May 26;16(5):e0251502. doi: 10.1371/journal.pone.0251502 (PMC8153501; doi:10.1371/journal.pone.0251502)
Supplement: S1 Table — (DOCX) [file pone.0251502.s005.docx]

**S1 Table.** Demographic and socioeconomic characteristics of the stratified sample aggregated by deaths by Substance Use Disorders (SUD) and other causes, 2005 to 2017

| **Risk Factor**  **(Level)** | **Individual deaths by SUD** | **Individual deaths by any other cause** | **Proportion SUD Deaths** |
| --- | --- | --- | --- |
| **Age Group** |  |  |  |
| <15 y | 23 | 3,571 | 0·64% |
| 15 – 19 y | 399 | 6,735 | 5·59% |
| 20 – 24 y | 1,652 | 11,048 | 13·01% |
| 25 – 29 y | 2,474 | 11,701 | 17·45% |
| 30 – 34 y | 2,612 | 12,884 | 16·86% |
| 35 – 39 y | 2,594 | 16,939 | 13·28% |
| 40 – 44 y | 2,706 | 26,012 | 9·42% |
| 45 – 49 y | 3,172 | 42,274 | 6·98% |
| 50 – 54 y | 2,977 | 67,130 | 4·25% |
| 55 – 59 y | 2,082 | 90,905 | 2·24% |
| 60 – 64 | 997 | 112,060 | 0·88% |
| > 64 y | 788 | 686,866 | 0·11% |
| Not Available | 7 | 591 | 1·17% |
| **Sex** |  |  |  |
| Females | 7,899 | 477,585 | 1·63% |
| Males | 14,584 | 611,131 | 2·33% |
| **Race** |  |  |  |
| White | 19,574 | 903,266 | 2·12% |
| Black | 2,460 | 152,579 | 1·59% |
| Other | 449 | 32,871 | 1·35% |
| **Educational Level** |  |  |  |
| Primary | 361 | 42,598 | 0·84% |
| Secondary | 2,789 | 142,454 | 1·92% |
| College-Level | 1,134 | 74,432 | 1·50% |
| Not Available | 18,199 | 829,232 | 2·15% |
| **Marital Status** |  |  |  |
| Never Married | 10,086 | 155,593 | 6·09% |
| Currently Married | 5,381 | 489,392 | 1·09% |
| Previously Married | 6,578 | 430,812 | 1·50% |
| Not Available | 438 | 12,919 | 3·28% |
| **Total** | **22,483** | **1,088,716** | **2**·**02%** |
