# Supplementary material for: “Know your epidemic, know your response”: Epidemiological assessment of the substance use disorder crisis in the United States
Source: PLoS One. 2021 May 26;16(5):e0251502. doi: 10.1371/journal.pone.0251502 (PMC8153501; doi:10.1371/journal.pone.0251502)
Supplement: S2 Table — (DOCX) [file pone.0251502.s006.docx]

**S2 Table.** Complementary results of the generalized additive model association analysis for the county level covariates with the estimated degrees of freedom (est. DoF) results for multilevel and factor smooth interaction models.

|  | | **Est. DoF** | | **Ref. est. DoF** | | **P-value** | |  | | |  |
| --- | --- | --- | --- | --- | --- | --- | --- | --- | --- | --- | --- |
| Percentage of uninsured population | | 3·240 | | 3·729 | | <0·001 | |  | | |  |
| Percentage of adults with excessive alcohol consumption | | 6·151 | | 6·653 | | <0·001 | |  | | |  |
| Percentage of adults consuming tobacco | | 1·001 | | 1·002 | | 0·403 | |  | | |  |
| Percentage of uninsured population | | 1·001 | | 1·002 | | 0·288 | |  | | |  |
| Average Mental Unhealthy Days | | 3·248 | | 3·772 | | <0·001 | |  | | |  |
| Average Physical Unhealthy Days | | 4·595 | | 5·187 | | <0·001 | |  | | |  |
|  | |  | |  | |  | |  | | |  |
| **FACTOR SMOOTHER INTERACTION MODEL** | | | | | | | | |  | | |
| **Mental Unhealthy Days by Age Group** | | | | | | | | |  | | |
|  | | **Est. DoF** | | **Ref. est. DoF** | | **P-value** | |  | | |  |
| >15 y | | 1·002 | | 1·004 | | 0·754 | |  | | |  |
| 15 – 19 y | | 1·003 | | 1·005 | | 0·162 | |  | | |  |
| 20 – 24 y | | 1·003 | | 1·005 | | 0·029 | |  | | |  |
| 25 – 29 y | | 1·008 | | 1·015 | | <0·001 | |  | | |  |
| 30 – 34 y | | 1·003 | | 1·005 | | <0·001 | |  | | |  |
| 35 – 39 y | | 1·966 | | 2·496 | | 0·004 | |  | | |  |
| 40 – 44 y | | 1·003 | | 1·005 | | <0·001 | |  | | |  |
| 45 – 49 y | | 1·002 | | 1·004 | | <0·001 | |  | | |  |
| 50 – 54 y | | 2·244 | | 2·863 | | 0·002 | |  | | |  |
| 55 – 59 y | | 2·336 | | 2·987 | | 0·352 | |  | | |  |
| 60 – 64 | | 1·004 | | 1·006 | | 0·451 | |  | | |  |
| < 64 y | | 1·002 | | 1·004 | | 0·544 | |  | | |  |
| Not Available | | 1·002 | | 1·004 | | 0·359 | |  | | |  |
| **Average Physical Unhealthy Days by Age Group** | | | | | | | | | | | |
| >15 y | 1·002 | | 1·004 | | 0·166 | |  | | |  |  |
| 15 – 19 y | 1·009 | | 1·018 | | 0·867 | |  | | |  |  |
| 20 – 24 y | 1·001 | | 1·003 | | 0·462 | |  | | |  |  |
| 25 – 29 y | 2·196 | | 2·784 | | 0·433 | |  | | |  |  |
| 30 – 34 y | 1·303 | | 1·542 | | 0·569 | |  | | |  |  |
| 35 – 39 y | 2·731 | | 3·413 | | 0·002 | |  | | |  |  |
| 40 – 44 y | 1·003 | | 1·005 | | 0·001 | |  | | |  |  |
| 45 – 49 y | 1·514 | | 1·860 | | 0·083 | |  | | |  |  |
| 50 – 54 y | 1·002 | | 1·004 | | 0·009 | |  | | |  |  |
| 55 – 59 y | 2·746 | | 3·465 | | <0·001 | |  | | |  |  |
| 60 – 64 | 2·655 | | 3·373 | | 0·001 | |  | | |  |  |
| < 64 y | 2·899 | | 3·678 | | <0·001 | |  | | |  |  |
| Not Available | 1·003 | | 1·007 | | 0·580 | |  | | |  |  |
| **Mental Unhealthy Days by Sex** | | | | | | | | | | | |
| Females | | 1·001 | | 1·002 | | <0·001 | |  | | |  |
| Males | | 4·109 | | 4·975 | | <0·001 | |  | | |  |
| **Physical Unhealthy Days by Sex** | | | | | | | | | | | |
| Females | | 1·001 | | 1·002 | | <0·001 | |  | | |  |
| Males | | 3·983 | | 4·817 | | 0·001 | |  | | |  |
| **Mental Unhealthy Days by Race** | | | | | | | | | | | |
| White | | 3·381 | | 4·002 | | <0·001 | |  | | |  |
| Black | | 1·003 | | 1·006 | | 0·998 | |  | | |  |
| Other | | 1·001 | | 1·002 | | 0·002 | |  | | |  |
| **Physical Unhealthy Days by Race** | | | | | | | | |  | | |
| White | | 4·089 | | 4·735 | | <0·001 | |  | | |  |
| Black | | 1·001 | | 1·001 | | 0·055 | |  | | |  |
| Other | | 3·060 | | 3·781 | | 0·106 | |  | | |  |
