# Supplementary material for: “Know your epidemic, know your response”: Epidemiological assessment of the substance use disorder crisis in the United States
Source: PLoS One. 2021 May 26;16(5):e0251502. doi: 10.1371/journal.pone.0251502 (PMC8153501; doi:10.1371/journal.pone.0251502)
Supplement: S3 Table — (DOCX) [file pone.0251502.s007.docx]

**S3 Table.** Identified clusters of deaths by substance use disorders from the U.S. individual mortality, 2005 to 2017.

| **CLUSTER** | **RADIUS** | **COUNTIES** | **OBSERVED SUD related deaths** | **EXPECTED SUD related Deaths** | **RELATIVE RISK** | **CHILDREN IN POVERTY (%)** | **UNINSURED POPULATION (%)** | **ADULTS EXCESSIVE ALCOHOL CONSUMPTION (%)** | **TOBACCO USE (%)** | **MENTALLY UNHEALTHY DAYS** | **PHYSICALLY UNHEALTHY DAYS** |
| --- | --- | --- | --- | --- | --- | --- | --- | --- | --- | --- | --- |
| 1 | 173·38 | 101 | 23,128 | 13,408 | 1·72 | 28·50 | 13·96 | 12·93 | 25·14 | 4·55 | 4·94 |
| 2 | 145·24 | 24 | 20,017 | 13,388 | 1·50 | 13·47 | 7·42 | 19·93 | 15·55 | 3·44 | 3·21 |
| 3 | 112·48 | 25 | 11,343 | 7,833 | 1·45 | 22·87 | 11·92 | 16·77 | 21·30 | 3·92 | 3·94 |
| 4 | 148·30 | 11 | 3,578 | 1,804 | 1·98 | 27·06 | 19·61 | 13·65 | 18·65 | 3·84 | 4·27 |
| 5 | 120·23 | 25 | 15,207 | 11,466 | 1·33 | 19·07 | 11·44 | 18·41 | 19·72 | 3·74 | 3·57 |
| 6 | 82·32 | 10 | 4,563 | 2,687 | 1·70 | 27·41 | 19·64 | 16·04 | 22·04 | 3·94 | 4·09 |
| 7 | 99·25 | 8 | 4,843 | 3,027 | 1·60 | 15·69 | 14·59 | 17·82 | 16·53 | 3·12 | 3·01 |
| 8 | 96·11 | 30 | 2,869 | 1,831 | 1·57 | 26·55 | 16·89 | 12·42 | 23·03 | 4·30 | 4·62 |
| 9 | 135·98 | 5 | 9,461 | 7,539 | 1·25 | 26·73 | 18·18 | 16·28 | 17·61 | 3·94 | 3·93 |
| 10 | 158·04 | 10 | 2,200 | 1,369 | 1·61 | 26·28 | 16·29 | 18·51 | 16·69 | 4·14 | 4·20 |
| 11 | 113·10 | 38 | 5,007 | 3,721 | 1·35 | 27·29 | 19·24 | 11·72 | 21·47 | 4·19 | 4·39 |
| 12 | 160·56 | 15 | 3,295 | 2,410 | 1·37 | 13·54 | 15·36 | 11·20 | 11·62 | 3·34 | 3·49 |
| 13 | 130·89 | 4 | 10,970 | 9,368 | 1·17 | 22·28 | 19·26 | 17·34 | 12·61 | 3·68 | 3·83 |
| 14 | 70·22 | 7 | 2,923 | 2,153 | 1·36 | 23·87 | 18·30 | 15·94 | 20·22 | 3·86 | 3·99 |
| 15 | 96·54 | 24 | 10,866 | 9,643 | 1·13 | 16·09 | 11·77 | 18·09 | 18·93 | 3·57 | 3·45 |
| 16 | 87·69 | 14 | 11,814 | 10,579 | 1·12 | 14·71 | 11·55 | 20·75 | 17·74 | 3·38 | 3·41 |
| 17 | 107·03 | 3 | 1,019 | 707 | 1·44 | 19·11 | 17·90 | 18·23 | 11·73 | 3·77 | 3·64 |
| 18 | 134·32 | 26 | 3,483 | 2,886 | 1·21 | 28·46 | 22·39 | 11·50 | 25·15 | 4·64 | 4·83 |
| 19 | 71·73 | 4 | 1,158 | 831 | 1·39 | 23·28 | 17·99 | 16·77 | 21·36 | 3·57 | 3·57 |
| 20 | 169·33 | 10 | 646 | 428 | 1·51 | 28·12 | 23·60 | 14·96 | 18·83 | 3·55 | 4·10 |
| 21 | 109·27 | 3 | 1,130 | 849 | 1·33 | 20·13 | 21·19 | 20·01 | 20·33 | 4·02 | 3·96 |
| 22 | 174·75 | 17 | 1,206 | 940 | 1·28 | 13·80 | 16·61 | 20·78 | 15·28 | 3·00 | 2·98 |
| 23 | 153·42 | 71 | 7,923 | 7,274 | 1·09 | 26·04 | 15·64 | 11·89 | 23·98 | 4·24 | 4·67 |
| 24 | 100·59 | 7 | 1,276 | 1,035 | 1·23 | 23·76 | 17·95 | 16·40 | 17·52 | 3·45 | 4·06 |
| 25 | 110·29 | 35 | 5,757 | 5,356 | 1·07 | 19·15 | 15·35 | 15·62 | 21·93 | 3·75 | 3·69 |
| **CLUSTERS** |  | **527** | **165,682** | **122,533** | **1·35** | **23·49** | **15·44** | **14·73** | **21·26** | **4·02** | **4·20** |
| **NO CLUSTERS** |  | **2,584** | **298,035** | **341,184** | **0·87** | **23·41** | **16·54** | **16·27** | **19·09** | **3·57** | **3·75** |
| **TOTAL** |  | **3,111** | **463,717** | **463,717** | **1·00** | **23·42** | **16·35** | **16·01** | **19·45** | **3·65** | **3·83** |
