# Supplementary material for: “Know your epidemic, know your response”: Epidemiological assessment of the substance use disorder crisis in the United States
Source: PLoS One. 2021 May 26;16(5):e0251502. doi: 10.1371/journal.pone.0251502 (PMC8153501; doi:10.1371/journal.pone.0251502)
Supplement: Attachment — Submitted filename: Response_Reviewers_Feb23.pdf [file pone.0251502.s008.pdf]

“Know your epidemic, know your response”: Epidemiological assessment of the substance use disorder crisis in the United States

Andrés Hernández, Minxuan Lan, Neil J. MacKinnon, Adam J. Branscum, Diego F. Cuadros

REPLY TO REVIEWERS’ COMMENTS

We appreciate the opportunity to resubmit our work to the journal. We would like to thank the editor and the reviewers for assessing our work and for their valuable feedback and suggestions that have improved our manuscript. Please find below a point-by-point reply that addresses each of the journal and the reviewers’ comments. We have also incorporated these suggestions in the revised manuscript as noted below. We would be pleased to address any further points that the editor or reviewers may find unsatisfactory.

JOURNAL COMMENTS:

1. Please ensure that your manuscript meets PLOS ONE's style requirements, including those for file naming.

Style requirements were followed in the revised version of the manuscript according to the guidelines provided by the journal, including the file naming patterns.

2. You indicated that ethical approval was not necessary for your study. We understand that the framework for ethical oversight requirements for studies of this type may differ depending on the setting and we would appreciate some further clarification regarding your research. Could you please provide further details on why your study is exempt from the need for approval and confirmation from your institutional review board or research ethics committee (e.g., in the form of a letter or email correspondence) that ethics review was not necessary for this study? Please include a copy of the correspondence as an "Other" file.

As we stated in our manuscript, we used for our analyzes the publicly available vital statistics micro-data from the Centers for Disease Control (CDC) compiled by the National Center for Health Statistics (NCHS) (https://www.cdc.gov/nchs/data_access/vitalstatsonline.htm). These data are neither identifiable nor private and thus do not meet the federal definition of “human subject” as defined in 45 CFR 46.102. Therefore, these research projects do not need to be reviewed and approved by an Institutional Review Board, (IRB). For your reference, a similar study using the same data sources as our study and published last year in the journal also stated that because their study involved analysis of existing, deidentified data, it was exempt from human subjects review (“Glei, Dana A., and Samuel H. Preston. "Estimating the impact of drug use on US mortality, 1999-2016." PloS one 15.1 (2020): e0226732.)

3. Please include captions for your Supporting Information files at the end of your manuscript, and update any in-text citations to match accordingly.

The supporting information captions were included at the end of the manuscript according to the style of the reference provided by the editor. (Page 20, Line 1, Supporting Information Section)

REVIEWER COMMENTS:

REVIEWER 1

Abstract:

1. For this sentence in the conclusion "we found that this sad epidemic in the U.S. is characterized by…" I got confused with "sad" standing for substance abuse disorder – perhaps change to SUD or rephrase

We appreciate the feedback of the reviewer. The term Substance Use Disorder (SUD) replaced all other related abbreviations along the manuscript for consistency, accordingly to the reviewer suggestion. (Page 2, line 3, Abstract section), (Page 2, line 16, Abstract section), (Page 8, line 16, Table 1 caption), (Page 8, line 18, Fig 1 caption), (Page 8, line 20, Fig 2 caption), (Page 10, line 4, Table 2 caption), (Page 10, line 7, Fig 3 caption), (Page 12, line 5, Fig 4 caption), (Page 13, line 2, Table 3 caption), (Page 12, line 5, Discussion section).

2. For the first sentence in the results section, consider spelling out "U.S." to United States so it is clearer that the next sentence begins with "white", otherwise this appears to be a run on sentence.

The suggestion was included in the reviewed version of the manuscript. (Page 2, line 11, Abstract section).

Introduction:

1. Regarding the definition of SUD, the manuscript states that the person that engages in the behavior has knowledge of the harmful consequences of engaging in excessive substance use. However, how is this measured and how do we know the individual is aware of the harm? In the research methods section the data collected is about substance use and behavior, not knowledge of the behavior.

We appreciate the feedback from the reviewer, and we agree with the reviewer about the need of further clarification of the definition of Substance Use Disorders (SUD) mortality. Our research methods focus on the analysis of unintentional (accidental) deaths by substance overdose as

defined by the ICD10 standard for causes of death codes X40, X41, X42, X43, X44. The other causes of deaths including codes X60, X61, X62, X63, X64 (Deaths by Intentional Self-harm/Suicide caused by substance overdose), and deaths with no information about the self-intention (codes Y10, Y11, Y12, Y13, Y14) were used as control events (outcome '0') in our binary outcome. This choice made the assumption that death by accidental substance overdose is a proxy for the mortality of substance abuse disorders that does not include the intention of self-harm. This choice was based on the literature review and implies some limitations worth noting. As a result of the reviewer suggestion, we included in the revised version of the manuscript a mention to this limitation as the data source does not specify previous medical history of deceased individuals. (Page 14, line 14, Discussion section, limitations).

2. End of page 3, states that "know your epidemic, know your response" framework shifted from coercive strategies to targeted prevention strategies. What does this mean? How were they coercive before, how did the shift occur to targeted prevention, and clarify what this means?

"Know your epidemic, know your response" is a framework that prioritize preventive approaches (e.g., education campaigns, targeting cause of epidemics) more than reactive measures (e.g., vaccination, medical treatment) for mitigating epidemics. In principle, this framework has been proposed to target HIV, however several studies reported that components of this approach are also applicable to other epidemics. Additionally, this framework states that the individual's knowledge about risk is critical for the ability to respond epidemics with risk reduction strategies, and the individual social behaviors are determinants on the prevalence of the epidemics. As a suggestion of the reviewer's feedback, we include an updated bibliography highlighting the aforementioned elements, and rephrasing the concept of coercive strategies for clarifying in the reviewed version of the manuscript. (Page 4, line 4, Introduction section).

Methods:

1. Page 5, second to last paragraph, last sentence should be "publicly" (not publicity)

We appreciate the careful examination of the reviewer. We included the suggestion in the reviewed version of the manuscript. (Page 5, line 20, Methods, Data source Description section).

Results:

1. Page 7, paragraph 1 – for the sentence that says mortality in Black males has risen sharply since 2014, 40.00 SUD-related deaths per 1,000 total deaths is listed, however I would like to see a comparison number for white males.

As a result of the reviewer's suggestion. We included in the revised version of the manuscript the comparison of the temporal trend of SUD-related deaths of black and white males. (Page 7, line 11, Results section).

2. Page 7 – table 1, figure 1 and figure 2 use the terminology substance abuse disorder (SAD) while the rest of the manuscript has been using substance use disorder (SUD) – clarification is needed here

We appreciate the feedback of the reviewer. The term Substance Use Disorder (SUD) was included along all the manuscript accordingly to the reviewer suggestion. (Page 2, line 3, Abstract section), (Page 2, line 16, Abstract section), (Page 8, line 16, Table 1 caption), (Page 8, line 18, Fig 1 caption), (Page 8, line 20, Fig 2 caption), (Page 10, line 4, Table 2 caption), (Page 10, line 7, Fig 3 caption), (Page 12, line 5, Fig 4 caption), (Page 13, line 2, Table 3 caption), (Page 12, line 5, Discussion section).

3. Page 8, paragraph 1 – states that there is no statistical difference in population odds of SUD-related death for males and females, this needs clarification as males suffer from disproportionately high SUD and have shown highest mortality 2005-2017 stated multiple places elsewhere.

Taking into account the suggestion of the reviewer, we added in the discussion section further mention about this result, and we included new bibliography that analyzed this topic with similar results as ours. Even though we agree with the reviewer that men suffer a disproportionately high SUD-related deaths, our association analysis resulted in a non-significant effect of gender on the risk of death by SUD likely caused by the relationship between gender, morbidity and other socioeconomic factors included in our adjusted model. (Page 13, line 14, Discussion section).

Discussion:

1. Middle paragraph on page 11 – the description of the epidemic in the southern Pacific/mountain region is a little unclear to me. 2-3 more sentences of elaboration would be helpful to contrast with the epidemic occurring in the Midwest and other regions.

As a result of the suggestion of the reviewer, we add more details and updated bibliography regarding the differences of the epidemic occurring in the Midwest and North East compared to the southern Pacific/mountain regions. (Page 14, line 2, Discussion section).

Figures:

1. Figure 2 – description of the figure and short analysis contrasting A and B would be helpful/useful

We added further comparison among the rates between black and white male population in Figure 2. We also rounded the morality rates to 2 decimals to compute the percentage of increasing rates for black and white males from 2014 to 2015. (Page 7, line 6, Abstract section).

2. Figure 4 – B – clarify 'intentional' substance use disorders

We appreciate the feedback of the reviewer and we agree with the need of clarification about the use of 'intentional' and 'unintentional' in the context of death by substance overdose. Our research methods include unintentional accidental deaths by substance overdose as defined by the ICD10 codes for causes of death X40, X41, X42, X43, X44. The other causes of deaths including codes X60, X61, X62, X63, X64 (Deaths by Intentional Self- harm/Suicide caused by substance overdose) and Y10, Y11, Y12, Y13, Y14 (Undetermined intent substance overdose death) were used as control cases in our binary outcome. This clarification is included in the reviewed version of the manuscript. (Page 14, line 14, Discussion section).

REVIEWER 2

1. Topic of considerable interest given Case and Deaton's PNAS paper and book on "Deaths of Despair" and the noted increased mortality due to suicide, overdose, accidents in select groups in the last 5-8 years.

We appreciate the interesting insights of the reviewer. We also agree that the reference the author is citing is an important piece of the literature related to our study, given the fact they used the same data source for the death counts (CDC Wonder micro-files), and validated some of our findings. As a result, we added this reference to our bibliographic review in the revised version of our manuscript and included more details to the discussion based on the findings of Case and Deaton's work. (Page 19, line 14, Reference section).

4. Results

Increase 25-29, higher education (Did they look at college/no college, single, divorced, poverty, physical illness? The opportunity to check out the Case and Deaton findings on education is partially lost by the absence of 2.18% of the data on education. The figures are presented for primary/secondary/college level. Can the authors look further at college graduate vs. others?

Our study reported the results from a statistical association analysis between the individual risk of death caused by unintentional substance poisoning and several demographic and socio-economic covariates, including (age group, gender, education, socio economic status and marital status), as well as self-reported days of physical and mental distress from the Behavioral Risk Factor Surveillance System averaged for the time period of 2010 – 2017 and aggregated at the county level.

From the results of our association analysis, we agree with the reviewer that Case and Deaton's research is an important piece of the literature to be discussed within the context of our study. However, Case and Deaton study shows several differences and similarities with our study that are worth noting because they limit our ability to compare both findings of both studies, especially in the relationship of educational level and SUD-related death risk. As a result of the reviewer's suggestion, we summarized the similarities and differences of our study with the Case and Deaton's work and included them in the revised version of our manuscript. (Page 13, Lines 10, Discussion section)

Among similarities and differences, Case and Deaton's research offers a wider range of death causes than the focus of our study. They also concentrated a significant portion of their study on midlife adults, and highlighted suicides, alcohol-related and overdose as one of the critical drivers of the mortality rates increase for whites non-hispanic (WNH) in the United States. In addition, they found that educational level was critical for several aspects of the death rate increment from 1999 - 2015. In our study, we excluded intentional self-harm, and other substances apart from (Heroin, Methadone, Other opioids, synthetic narcotics, cocaine, unspecified narcotics) and included a wider range of age groups (from 5 to 84 years). As age is related to educational level, this could explain that college education did not show statistical significance, but high school resulted in an associated factor for the increased risk of SUD-related death.

Another difference is the time range of both studies. While Case and Deaton's focused from 1999 to 2015, our study was conducted from 2005 to 2017. This is especially important because Case and Deaton study reported an increase of WNH death rates from 1999 to 2015, and a parallel decrease of death rates for Blacks and Hispanic population on the same period. Our study shows that Black SUD-related death rates rised sharply from 2013 and reach the WNH SUD-related death rates in 2017, period which was out of the scope of the Case and Deaton's study.

Finally, Case and Deaton were similar to our methods in the use of the same data sources for the computation of mortality rates, and a resulting increase of morbidity of physical and mental health linked to the increase of death rates. This offers an interesting insight to our study because they exposed the lack of job opportunities and education as a potential cause of the increasing death rates. In addition, they validated our spatiotemporal pattern which suggest a diffusion of the epidemic from the Southwest to the North Eastern and Mideastern states and an unclear association between gender and wealth with an increasing of alcohol, suicide and overdose related death rates that is a super set of the SUD-related mortality rates of our study.
